# Functional Equivalence of Retroviral MA Domains in Facilitating Psi RNA Binding Specificity by Gag

**DOI:** 10.3390/v8090256

**Published:** 2016-09-19

**Authors:** Tiffiny Rye-McCurdy, Erik D. Olson, Shuohui Liu, Christiana Binkley, Joshua-Paolo Reyes, Brian R. Thompson, John M. Flanagan, Leslie J. Parent, Karin Musier-Forsyth

**Affiliations:** 1 Department of Chemistry and Biochemistry, The Ohio State University, Center for Retroviral Research, and Center for RNA Biology, Columbus, OH 43210, USA; rye.10@osu.edu (T.R.-M.); olson.249@osu.edu (E.D.O.); liu.4982@buckeyemail.osu.edu (S.L.); binkley.94@osu.edu (C.B.); reyes.313@buckeyemail.osu.edu (J.-P.R.); thompson.2255@buckeyemail.osu.edu (B.R.T.); 2 Department of Biochemistry and Molecular Biology, Penn State College of Medicine, Hershey, PA 17033, USA; jmf27@psu.edu; 3 Departments of Medicine and Microbiology and Immunology, Division of Infectious Diseases and Epidemiology, Penn State College of Medicine, Hershey, PA 17033, USA; lparent@psu.edu

**Keywords:** Rous sarcoma virus (RSV), human immunodeficiency virus type 1 (HIV-1), Gag, Psi RNA (Ψ RNA), matrix (MA), nucleocapsid (NC)

## Abstract

Retroviruses specifically package full-length, dimeric genomic RNA (gRNA) even in the presence of a vast excess of cellular RNA. The “psi” (Ψ) element within the 5′-untranslated region (5′UTR) of gRNA is critical for packaging through interaction with the nucleocapsid (NC) domain of Gag. However, in vitro Gag binding affinity for Ψ versus non-Ψ RNAs is not significantly different. Previous salt-titration binding assays revealed that human immunodeficiency virus type 1 (HIV-1) Gag bound to Ψ RNA with high specificity and relatively few charge interactions, whereas binding to non-Ψ RNA was less specific and involved more electrostatic interactions. The NC domain was critical for specific Ψ binding, but surprisingly, a Gag mutant lacking the matrix (MA) domain was less effective at discriminating Ψ from non-Ψ RNA. We now find that Rous sarcoma virus (RSV) Gag also effectively discriminates RSV Ψ from non-Ψ RNA in a MA-dependent manner. Interestingly, Gag chimeras, wherein the HIV-1 and RSV MA domains were swapped, maintained high binding specificity to cognate Ψ RNAs. Using Ψ RNA mutant constructs, determinants responsible for promoting high Gag binding specificity were identified in both systems. Taken together, these studies reveal the functional equivalence of HIV-1 and RSV MA domains in facilitating Ψ RNA selectivity by Gag, as well as Ψ elements that promote this selectivity.

## 1. Introduction

**Figure 1:**
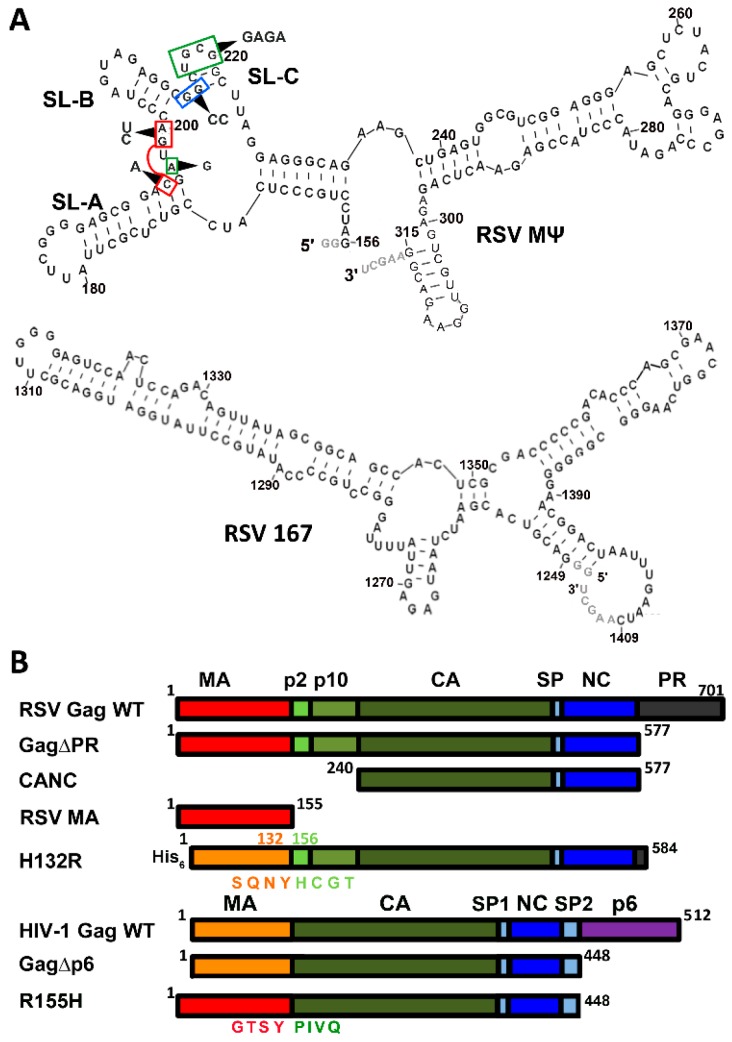
Rous sarcoma virus (RSV) RNA, and RSV and human immunodeficiency virus type 1 (HIV-1) protein constructs used in this work. (**A**) Predicted secondary structures of RSV MΨ (**top**) and RSV 167 (**bottom**) derived from nucleotides (nt) 156–315 and 1249–1409 of the RSV Prague C genome, respectively. Four mutant MΨ constructs are indicated by the boxed nt and arrows: UGCG to GAGA, GG to CC, A197G, and a triple mutation AGC to UCA. Additional nt at the 5′ and 3′ ends of each RNA that are not present in the RSV genome are shown in gray; (**B**) RSV and HIV-1 Gag constructs used in this work, with full-length wild-type Gag shown at the top of each set for comparison. Matrix (MA), capsid (CA) and nucleocapsid (NC), as well as various spacer peptides (SP, SP1, and SP2), the HIV-1 p6 domain, and the RSV p2, p10, and protease domain (PR) comprise these constructs. In the case of the two chimeric constructs, H132R and R155H, the residues at the junctions are explicitly shown. H132R contains the 132-residue HIV-1 MA in place of RSV MA in the context of RSV Gag, and R155H contains the 155-residue RSV MA in place of HIV-1 MA in the context of HIV-1 Gag.

A hallmark of retroviruses is their ability to reverse transcribe single-stranded genomic RNA (gRNA) into double-stranded DNA for subsequent integration into the host cell genome. Following nuclear transcription and export into the cytoplasm, highly selective packaging of the full-length, unspliced gRNA into newly assembled virus particles is achieved, despite the presence of spliced viral RNA and a vast excess of cellular RNA [1,2]. Human immunodeficiency virus type 1 (HIV-1) packages only a single, dimeric copy of its gRNA [3,4,5,6]. Specific HIV-1 gRNA packaging is achieved, in part, via the interaction between the nucleocapsid (NC) domain of the HIV-1 Gag polyprotein and the “psi” (Ψ) packaging signal, located in the gRNA 5′-untranslated region (5′UTR) [7,8]. In addition to the NC domain, HIV-1 Gag consists of matrix (MA), capsid (CA), two spacer peptides, and p6 (Figure 1B). In the context of the polyprotein, MA targets the assembling virus to the plasma membrane (PM) [9], CA is involved in Gag multimerization associated with immature viral particle formation [10,11], NC functions as a nucleic acid chaperone in addition to its role in gRNA packaging [12,13,14,15,16,17,18], and the p6 domain recruits factors required for viral fission from the cell [19,20]. Importantly, Gag’s main structural domains provide functional redundancy to ensure proper viral assembly through Gag–nucleic acid, Gag–Gag, and Gag–membrane interactions [21,22].

Although the minimal element that is both necessary and sufficient to confer selective HIV-1 gRNA packaging is not yet known, a gRNA packaging competition assay has defined a “core encapsidation signal” composed of the U5 region and stem-loops 1–4 (SL1–SL4) of Ψ [7]. In the absence of Ψ, the Gag polyprotein can still direct efficient production of virus-like particles and cellular RNAs are incorporated into particles at levels corresponding to their relative amounts in the cytoplasm [23]. This finding indicates that Ψ provides a “packaging advantage” to gRNA, and in the absence of this signal, random RNAs are passively incorporated into viral particles. Within the stem-loops that constitute Ψ, exposed G residues in loops and bulges have been shown to be high-affinity NC binding sites based on a number of lines of evidence including selective 2′-hydroxyl analyzed by primer extension (SHAPE) footprinting studies of gRNA in virions [24], in vitro RNA binding and footprinting assays [25,26], and crosslinking-immunoprecipitation-sequencing (CLIP-seq) techniques in HIV-1 infected cells [8].

Several factors in addition to Gag/NC binding affinity for Ψ have recently been proposed to play a role in selective gRNA packaging. In vitro fluorescence-anisotropy (FA) salt-titration binding assays showed that HIV-1 Gag binding to RNA derived from the gRNA Ψ region is characterized by highly specific non-electrostatic binding and an effective charge that is consistent with an NC-only binding mode [27]. In contrast, binding to a non-Ψ RNA is characterized by less specific binding and a higher effective charge, consistent with nucleic acid binding by both NC and MA domains. Thus, changes in Gag’s binding conformation may contribute to the high degree of discrimination between Ψ and non-Ψ RNA [27].

In the case of Rous sarcoma virus (RSV), nucleotides (nt) 156–315 in the 5′UTR of the gRNA (designated as MΨ) can direct packaging of heterologous RNAs with an efficiency similar to that of the full-length gRNA [28,29]. Subsequent work showed that an 82-nt sequence (nt 156–237) containing three stem-loops, SL-A, SL-B, and SL-C, and referred to as μΨ, is the minimal sequence required for efficient gRNA packaging in RSV [30,31]. Similar to HIV-1, RSV Gag contains the MA, CA, and NC domains, all responsible for similar functions as in HIV-1 (Figure 1B) [32,33,34,35,36,37,38,39]. Distinct from HIV-1, RSV Gag contains the p2, p10, and protease (PR) domains, involved in budding [40], virion core structure and nuclear export [41], and virus particle maturation [42], respectively. Similar to HIV-1, interactions between RSV NC and the Ψ element in gRNA are believed to be responsible for specific gRNA packaging [35,36,37]. Cell-based assays revealed the importance of the linker sequence (nt 197–200) between SL-A and SL-B in gRNA packaging [30,43], and an A197G mutant severely inhibited RSV NC binding [44]. A nuclear magnetic resonance spectroscopy (NMR) study revealed interactions between residues in the C-terminal zinc finger and A197 [45]. A hydrophobic pocket in the N-terminal zinc finger of RSV NC was shown to interact with G residues in the UGCG tetraloop of SL-C via stacking interactions, and mutation of the SL-C tetraloop to GAGA abolished high-affinity NC binding in vitro [44]. Mutation of UGCG to GAGA decreased viral infectivity and A197G abolished infectivity completely [45]. In contrast, a 2-nt SL-C loop (GC to UU) mutation did not impact gRNA packaging efficiency in vivo, whereas stem mutations did have an impact [30].

As mentioned earlier, previous FA salt-titration binding assays provided insights into specific Ψ RNA recognition by HIV-1 Gag [27]. Two key parameters are obtained using this approach, *K*_d(1M)_, which describes the non-electrostatic component of binding (i.e., the extrapolated dissociation constant at 1 M salt), and *Z*_eff_, the effective charge of the protein–nucleic acid interaction, which is the number of Na^+^ cations displaced upon nucleic acid binding [46]. We previously reported that HIV-1 Gag binds to Ψ RNA with a dramatically reduced *K*_d(1M)_ and lower *Z*_eff_ relative to a non-Ψ RNA (TARpolyA). These results suggest that Gag interacts with RNA using different binding modes; both the NC and MA domains bind to RNA in the case of TARpolyA, whereas binding to Ψ RNA preferentially involves the NC domain at the expense of MA interactions. Mutations altering the NC zinc finger motifs of Gag significantly reduce the non-electrostatic component of binding to Ψ RNA and lead to an increase in *Z*_eff_. Surprisingly, the specific binding of Gag to Ψ also depended on the presence of the MA domain, as a Gag variant lacking MA was less able to discriminate Ψ from non-Ψ RNA and bound both RNAs with similar overall charge [27].

Although less is known about specific Ψ RNA recognition and packaging for RSV, the simplicity of the RSV Ψ element makes it an attractive model to further examine the mechanism by which Gag binds Ψ RNA in vitro and to investigate the role of MA in gRNA selection. We previously compared the nucleic acid chaperone functions of RSV and HIV-1 Gag and found that both proteins display robust NC domain-dependent chaperone activity in vitro [16,47]. In the case of HIV-1, inositol phosphates (IPs) stimulate Gag’s chaperone activity by binding to the MA domain [16], whereas IPs do not regulate the chaperone function of RSV Gag [47]. This mechanistic difference was determined to be due to differences in the MA domains. In the present work, we used the FA salt-titration assay to investigate recombinant RSV Gag binding to wild-type (WT) and mutant Ψ and non-Ψ RNAs for the first time, and probed the role of MA in binding specificity. Studies using chimeric Gag constructs revealed that RSV and HIV-1 MA domains were functionally interchangeable in this in vitro assay, and both contributed to selective recognition of Ψ RNA by Gag.

## 2. Materials and Methods

### 2.1. Preparation of Proteins and Nucleic Acids

All recombinant proteins were expressed and purified from *Escherichia coli*. The RSV Gag∆PR construct, pET28(-His).RSVGag∆PR, derived from the Prague C strain of RSV [48,49], contains MA through NC of the RSV Gag polyprotein [50] (Figure 1B). The R155H chimera was prepared by amplifying RSV MA from pET28(-His).RSVGag∆PR and cloning it in place of HIV-1 MA within the HIV-1 Gag∆p6 construct using standard PCR methods. RSV Gag∆PR and R155H were purified using the same method. Briefly, protein expression was induced in BL21(DE3) cells using 1 mM isopropyl β-d-1-thiogalactopyranoside (IPTG, Roche Life Sciences, Indianapolis, IN, USA) at 37 °C for 4 h; pellets were homogenized in lysis buffer (50 mM Tris HCl, pH 7.4, 700 mM NaCl, 0.1 mM 2-mercaptoethanol (βME), 10% glycerol, 0.1% triton X-100, and 1 cOmplete™ Mini ethylenediaminetetraacetic acid (EDTA)-free Protease Inhibitor Cocktail Tablet (Roche Life Sciences,), followed by addition of 120 kilounits of Ready-Lyse™ Lysozyme Solution (Epicentre, Madison, WI, USA) to the suspension and stirring on ice for 10 min. The resulting solution was polyethylenimine (Sigma-Aldrich, St. Louis, MO, USA) treated to remove nucleic acids, followed by ammonium sulfate precipitation, as previously described [47]. RSV Gag∆PR and R155H were then isolated via fibrous cellulose phosphate, as described previously [51], and fractions containing protein were further purified on a Superdex 200 column (GE Healthcare, Pittsburgh, PA, USA) run in 10 mM 4-(2-hydroxyethyl)-1-piperazineethanesulfonic acid (HEPES) (Fisher Scientific, Waltham, MA, USA), pH 7.4, 500 mM NaCl, 0.1 mM ZnCl_2_, 0.1 mM EDTA, and 1 mM βME.

To generate the plasmid encoding RSV CANC, PCR primers were designed to amplify the DNA encoding from the first residue of CA (proline) through the last residue of NC (serine) from pET28.(-His).Gag∆PR. The product was then subcloned into the NdeI and HindIII sites of pET28.(-His).Gag∆PR, replacing Gag∆PR with CANC and generating pET28.(-His).CANC. RSV CANC was purified essentially as described for RSV Gag∆PR with the following changes. The protein was expressed in BL21(DE3)-pRIL cells using autoinduction media, and a sulfopropyl sepharose column (GE Healthcare) was used in lieu of cellulose phosphate in buffer containing 25 mM HEPES, pH 7.5, 0.1 mM tris(2-carboxyethyl)phosphine (TCEP), 0.1 mM EDTA, and a stepwise gradient of NaCl.

The plasmid encoding HIV-1 Gag∆p6 was a gift from Dr. Alan Rein (HIV Dynamics and Replication Program, Center for Cancer Research, National Cancer Institute, Frederick, MD, USA). HIV-1 Gag∆p6 was expressed and purified using established methods [52]. H6.H132R.3h (hereafter referred to as H132R) and RSV MA were purified as previously described [47]. All protein concentrations were determined from the absorbance at 280 nm using the following molar extinction coefficients: 63,348 M^−1^·cm^−1^ (RSV Gag∆PR), 63,090 M^−1^·cm^−1^ (HIV-1 Gag∆p6), 67.295 M^−1^·cm^−1^ (R155H), 59,275 M^−1^·cm^−1^ (H132R), 21,095 M^−1^·cm^−1^ (RSV MA), and 33,646 M^−1^·cm^−1^ (RSV CANC).

All RNA constructs were in vitro transcribed from linearized plasmids using T7 RNA polymerase and previously established methods [53] (Figure 1A and Figure 5A). The DNA sequences encoding the RSV RNAs were synthesized and cloned into pIDTSMART-AMP by Integrated DNA Technologies (Coralville, IA, USA). The RSV 167 RNA construct was derived from nt 1249–1409 and RSV MΨ WT and mutant RNA constructs were derived from nt 156–315 of RSV Prague C strain, respectively. Each RNA was designed with two non-genomic G residues at the 5′ end to promote T7 polymerase transcription and AAGCU is added to the 3′ end as a result of the HindIII restriction enzyme used to linearize the plasmid prior to transcription. RSV MΨ A197G, MΨ GG to CC (G214C, G215C), MΨ Triple (C195A, G199C, A200U), and MΨ UGCG to GAGA (U217G, G218A, C219G, G220A) were prepared from the RSV RNA MΨ WT plasmid via site-directed mutagenesis using overlap extension PCR [54]. The HIV-1 Ψ-WT RNA construct was derived from nt 229–333 and the TARpolyA RNA construct was derived from nt 1–104 of the HIV-1 NL4-3 genome [55]. Plasmids encoding both RNAs contain FokI restriction enzyme cut sites to generate the correct 3′ end of the RNA. All HIV-1 Ψ RNA variants were designed with 2 additional G residues at the 5′ end to promote efficient T7-mediated transcription. HIV-1 Ψ-Mut 1 (G241,242A), Ψ-Mut 2 (G274,275A), Ψ-Mut 3 (G292A, U293A, G294A), Ψ-Mut 4 (G311A), Ψ-Mut 5 (G319A, G321A), Ψ-Mut 6 (G329,330A), Ψ-Mut 7 (G235A), and Ψ-Mut 8 (G267,268A) were prepared from the HIV-1 Ψ-WT template plasmid using QuikChange (Agilent Technologies, Santa Clara, CA, USA) or site-directed ligase-independent mutagenesis (SLIM) [56].

### 2.2. FA-Based Salt Titration Assays

All RNAs were labeled with fluorescein-5-thiosemicarbazide (FTSC) at the 3′ as described [57]. The concentration and labeling efficiency were determined by measuring the absorbance at 260 nm and 495 nm and using the following molar extinction coefficients: ε_495nm_ = 8.5 × 10^4^ M^−1^·cm^−1^ (fluorescein), ε_260nm_ = 1.47 × 10^6^ M^−1^·cm^−1^ (RSV MΨ WT, RSV MΨ mutants, and RSV 167), ε_260nm_ = 9.7 × 10^5^ M^−1^·cm^−1^ (HIV-1 Ψ WT and HIV-1 Ψ mutants), and ε_260nm_ = 9.3 × 10^5^ M^−1^·cm^−1^ (HIV-1 TARpolyA). Direct FA binding assays were performed at 20 mM HEPES pH 7.5, 1 mM MgCl_2_, 50 mM NaCl, and 1.5 nM RNA to obtain a preliminary assessment of binding affinity and to establish the concentration of each protein to use in the salt-titration studies as previously described [27,46]. FA salt-titration binding assays were carried out using established protocols [27,46]. Assays were performed with 1.5–20.5 nM RNA (as indicated in the figures and tables) in 20 mM HEPES or Tris-HCl, pH 7.5, 1 mM MgCl_2_, and 50 mM monovalent ions with 25 µM RSV MA, 300–400 nM HIV-1 Gag∆p6, 300 nM RSV CANC, 400 nM R155H, 300 nM H132R, and 300–500 nM RSV Gag∆PR. All fluorescence measurements were performed on a SpectraMax M5 plate reader (Molecular Devices, Sunnyvale, CA, USA).

## 3. Results

### 3.1. RSV Gag∆PR Specificity for Ψ RNA Requires the MA Domain

The RSV MΨ construct studied here corresponds to the previously identified packaging signal, and is sufficient to promote efficient gRNA packaging in RSV [30,31] (Figure 1A). RSV 167 is a non-Ψ RNA construct of the same length as MΨ (167 nt), derived from a region within the *gag* gene that has no known role in RSV gRNA packaging (Figure 1A). To test the relative binding affinity of RSV Gag∆PR and other RSV Gag constructs (Figure 1B) for Ψ and non-Ψ RNAs, direct FA binding assays were first performed with RSV MΨ and RSV 167 in 50 mM monovalent ions. Under these conditions, RSV Gag∆PR bound to RSV 167 and RSV MΨ RNAs with a similar affinity (*K*_d_ = 15–18 nM) (Table S1). Similarly, RSV CANC, which lacks MA, p2, p10, and PR, bound both RNAs with similar affinities (*K*_d_ ~ 11–21 nM) (Table S1). In contrast, RSV Gag∆NC and RSV MA bound RSV 167 and RSV MΨ RNAs very weakly, with *K*_d_ values ranging from ~1 to 6 µM (Table S1). Taken together with our previous work [47], these results confirm that NC is the primary RNA binding domain in RSV Gag, but that differences in binding affinity alone are not sufficient to explain the capability of RSV Gag to incorporate Ψ-containing RNA into virions with such high specificity.

**Figure 2:**
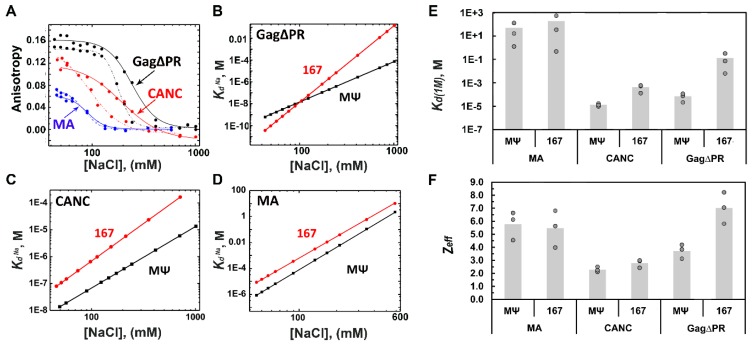
(**A**) Salt dependence of RSV Gag∆PR, RSV CANC (construct lacking RSV MA, p2, p10, and PR), and RSV MA binding to RSV MΨ (solid curves) and RSV 167 (dashed curves). Data from panel A are re-graphed in log-log plots of the apparent binding affinity (*K*_d_) as a function of NaCl concentration for RSV Gag∆PR (**B**); RSV CANC (**C**); and RSV MA (**D**); Bar graphs of *K*_d(1M)_ values (**E**) and *Z*_eff_ values (**F**) were determined by salt-titration assays using the indicated RSV proteins and RNAs. *K*_d(1M)_ values describe the nonelectrostatic component of binding and *Z*_eff_ values describe the electrostatic component of the protein–nucleic acid interactions [46]. Values of three trials performed in each case are shown with the height of the bar indicating the mean value.

To further test whether there are any mechanistic differences in RSV Gag∆PR interaction with Ψ versus non-Ψ RNAs, the salt dependence of the apparent binding affinity was measured, allowing contributions from both electrostatic and nonelectrostatic interactions to be assessed [46]. RSV Gag∆PR binding to RSV MΨ was characterized by a ~1900-fold stronger nonelectrostatic binding component (*K*_d(1M)_ = 7.1 × 10^−5^ M) and 3 fewer electrostatic interactions (*Z*_eff_ ~ 4) than binding to RSV 167 (*K*_d(1M)_ = 1.3 × 10^−1^ M and *Z*_eff_ = 7) (Figure 2 and Table 1). These values are comparable to those previously obtained for HIV-1 Gag∆p6 binding to HIV-1 Ψ versus TARpolyA (a non-Ψ RNA) (Table 1) [27]. Interestingly, RSV CANC binds to RSV MΨ with only a ~30-fold stronger nonelectrostatic binding component (*K*_d(1M)_ = 1.3 × 10^−5^ M) and the same number of electrostatic interactions (*Z*_eff_ = 2.3) as RSV 167 (*K*_d(1M)_ = 4.2 × 10^−4^ M and *Z*_eff_ = 2.8), suggesting that the MA domain is responsible for the three additional charge contacts made between Gag∆PR and non-Ψ RNA. This finding is consistent with a previous study showing that HIV-1 MA contributes to Ψ versus non-Ψ RNA discrimination by HIV-1 Gag∆p6 (Table 1) [27]. While we cannot rule out a contribution from the p2 and p10 domains not present in the CANC construct, these peptides lack basic character and are unlikely to interact with nucleic acids. To determine whether RSV MA intrinsically contributes to Gag specificity for Ψ RNA, salt-titration binding assays were performed using purified RSV MA. The RSV MA domain lacked binding specificity for RSV MΨ (*K*_d(1M)_ = 4.9 × 10^+1^ M and *Z*_eff_ ~ 6) and bound RSV 167 with very similar affinity and charge interactions (*K*_d(1M)_ = 1.9 × 10^+2^ M and *Z*_eff_ ~ 6) (Figure 2 and Table 1). HIV-1 MA generally binds RNA with much higher affinity than RSV MA [16,47,58] but also fails to discriminate between HIV-1 Ψ and TARpolyA RNAs based on FA salt-titration assay results [59]. Taken together, these and previous data suggest that both HIV-1 Gag∆p6 and RSV Gag∆PR interact with Ψ RNA in an NC-only binding mode using more nonelectrostatic contacts and with fewer charge interactions than binding to non-Ψ RNAs. The latter binding interaction involves additional electrostatic contacts, most likely with the MA domain. More importantly, MA is required for Gag-facilitated high-specificity binding to Ψ RNA in both retroviruses.

### 3.2. Retroviral MA Domains Are Functionally Equivalent in Facilitating Ψ versus Non-Ψ RNA Discrimination by Gag

The mechanism by which MA contributes to Ψ-binding specificity in the context of the Gag polyprotein is not known. To begin to address this question, two protein chimeras were investigated wherein MA domains of HIV-1 and RSV Gag were swapped. In H132R, the RSV MA domain is replaced by the 132-residue HIV-1 MA domain, whereas in R155H, HIV-1 MA is replaced by the 155-residue RSV MA domain (Figure 1B). Salt-titration binding assays were then performed using RSV Gag∆PR, HIV-1 Gag∆p6, H132R, and R155H with RSV MΨ and RSV 167 RNAs (Figure 1A), as well as with HIV-1-derived RNAs (Ψ and TARpolyA, Figure 5A). The latter RNAs are similar to constructs used previously in studies with HIV-1 Gag∆p6 [27]. In this previous work, we used higher RNA concentrations (~20–40 nM) in the salt-titration binding assay than used in the experiments with RSV Gag∆PR reported here. To arrive at the final RNA concentrations used in these assays, we tested a range of Ψ and non-Ψ RNA concentrations (1.5–20.5 nM) in the salt-titration assay with cognate Gag proteins (Table S2 and Figure S1). We determined that HIV-1 Gag∆p6 binds with the highest specificity at the highest RNA concentrations tested (20.5 nM, 1:20 RNA:protein ratio) and binds with low specificity to Ψ at the lowest RNA concentrations tested (1.5 nM, 1:200 RNA:protein ratio). In contrast, RSV Gag∆PR displayed the opposite trend, showing the greatest specificity for MΨ RNA at 1.5 nM RNA (1:200 RNA:protein ratio), although specific binding was still maintained at 20.5 nM RNA (1:20 RNA:protein ratio). All further salt titrations were performed at the RNA concentration at which the greatest binding specificity for cognate Ψ RNA was observed for each retroviral Gag protein.

**Figure 3:**
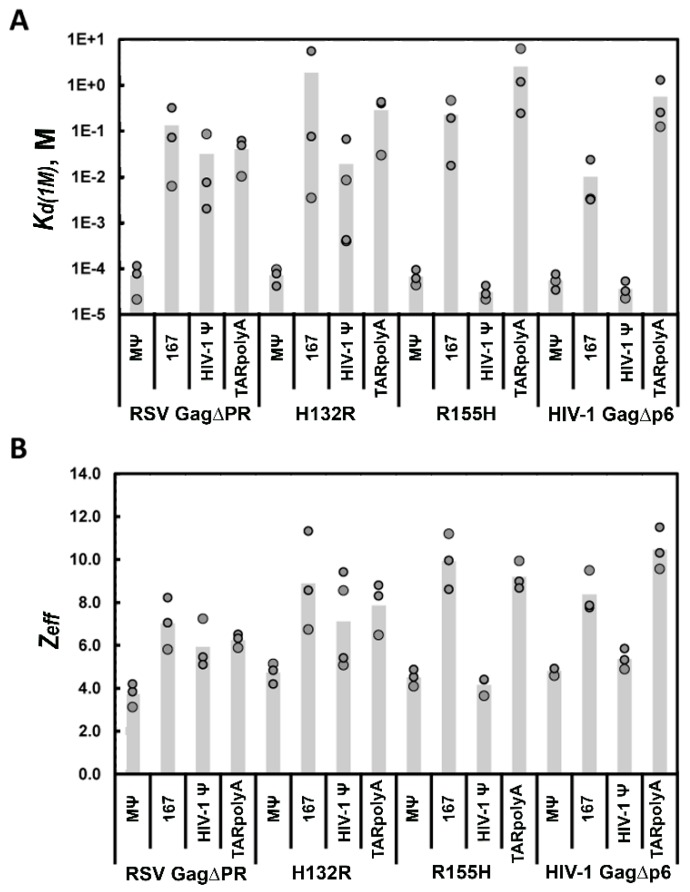
Bar graphs showing *K*_d(1M)_ values (**A**) and *Z*_eff_ values (**B**) determined from salt-titration assays with RSV Gag∆PR, H132R, R155H, and HIV-1 Gag∆p6 with RSV MΨ, RSV 167, HIV-1 Ψ, and HIV-1 TARpolyA. Values of three or four trials performed in each case are shown with the height of the bar indicating the mean value.

The chimeric H132R Gag construct interacted with RSV MΨ with a highly specific *K*_d(1M)_ = 7.2 × 10^−5^ M, which was very similar to RSV Gag∆PR, whereas the *Z*_eff_ was slightly higher at ~5 (versus ~4) (Figure 3 and Table 2). Furthermore, similar to RSV Gag∆PR, H132R interacted with RSV 167 nonspecifically with a *K*_d(1M)_ ~ 1.9 M and a large *Z*_eff_ of ~9. These data suggest that despite the presence of a heterologous HIV-1 MA domain, RSV Gag was still able to distinguish Ψ from non-Ψ RNA. In the reciprocal experiment, R155H maintained a specific *K*_d(1M)_ = 3.1 × 10^−5^ M with HIV-1 Ψ RNA and a low *Z*_eff_ of ~4, while the chimera’s interaction with HIV-1 TARpolyA was characterized by a nonspecific *K*_d(1M)_ ~ 2.6 M and high *Z*_eff_ of ~9 (Figure 3 and Table 2). Thus, HIV-1 Gag was also capable of accommodating another retroviral MA domain while still retaining the ability to differentiate Ψ from non-Ψ RNA. Taken together, these data indicate an important, but non-specific role for MA in Ψ recognition, with RNA specificity likely arising from the identity of the NC domain.

We next examined whether the WT and chimeric Gag constructs could specifically interact with the heterologous Ψ RNA. RSV Gag∆PR bound HIV-1 Ψ and TARpolyA with a *K*_d(1M)_ value of 3.2 × 10^−2^ M and 4.0 × 10^−2^ M, respectively, and a *Z*_eff_ ~ 6 for both RNAs, indicating that RSV Gag was unable to bind specifically to HIV-1 Ψ (Figure 3 and Table 2). Similarly, H132R bound HIV-1 Ψ and TARpolyA RNAs with a *K*_d(1M)_ value of 1.9 × 10^−2^ M and 2.9 × 10^−1^ M, respectively, and a *Z*_eff_ value of 7 and 8, respectively (Figure 3 and Table 2). In contrast, HIV-1 Gag∆p6 bound RSV MΨ in a specific manner with a *K*_d(1M)_ = 5.5 × 10^−5^ M and *Z*_eff_ ~ 5, and bound 167 more electrostatically with a *K*_d(1M)_ = 1.0 × 10^−2^ M and *Z*_eff_ ~ 8 (Figure 3 and Table 2). R155H behaved similarly to HIV-1 Gag∆p6 in its ability to distinguish RSV Ψ RNA, interacting with RSV MΨ with a specific *K*_d(1M)_ = 6.7 × 10^−5^ M and *Z*_eff_ ~ 5, whereas the RSV 167 interaction is characterized by a nonspecific *K*_d(1M)_ = 2.3 × 10^−1^ M and *Z*_eff_ ~ 10 (Figure 3 and Table 2). Taken together, these results indicate that, although the presence of a retroviral MA domain facilitated specific Ψ RNA binding, the identity of the MA domain did not confer specific recognition of a particular Ψ RNA sequence. That is, recognition of the chimeras followed the same pattern as the respective WT Gag constructs, supporting the conclusion that the NC domain determines the RNA specificity of Gag, while the role of MA in this mechanism appears to be purely non-specific/electrostatic in nature.

### 3.3. Mutations of RSV Ψ RNA Important for NC Binding Do Not Contribute to Specific Recognition by RSV Gag∆PR

**Figure 4:**
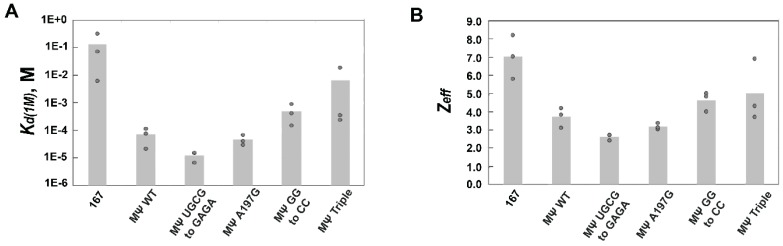
Bar graphs of *K*_d(1M)_ values (**A**) and *Z*_eff_ values (**B**) determined from salt-titration assays with RSV Gag∆PR and RSV 167, WT MΨ, and MΨ RNA mutants. Values of three trials performed in each case are shown with the height of the bar indicating the mean value.

We next wanted to investigate the Ψ RNA elements required for specific recognition by Gag. In RSV, it was previously reported that mutations A197G and UGCG to GAGA (nt 217–220) in RSV µΨ (nt 156–237, Figure 1A) lead to a 1500 and 10,000-fold loss in binding affinity to RSV NC, respectively [30,44]. A separate report demonstrated that mutations GG to CC (nt 214 and 215) and a triple mutant of nt 195, 199, and 200 in RSV MΨ (hereafter referred to as MΨ Triple) (Figure 1A) lead to 20- and 50-fold losses in gRNA packaging efficiency, respectively [30]. We first tested direct binding by RSV Gag∆PR for these RSV MΨ RNA mutant constructs. All *K*_d_ values were measured to be between ~13 and 18 nM (Table S3); thus, differences in binding affinity alone were unable to explain the previously reported results. To further examine how these mutations affect RSV Gag selectivity for Ψ RNA, FA salt titration assays were performed. RSV Gag∆PR bound both RSV MΨ A197G and MΨ UGCG to GAGA with specific *K*_d(1M)_ values of ~10^−5^ M and low *Z*_eff_, values (~3) that were essentially indistinguishable from binding to WT Ψ (Figure 4 and Table 3). Based on the *K*_d(1M)_ values, binding to these mutant RNAs was actually more specific (~1.5–6-fold) than binding to WT Ψ RNA.

In contrast, RSV MΨ GG to CC and MΨ Triple RNAs led to a loss in specific recognition by RSV Gag∆PR. The relative specificity decreased by ~seven-fold for the double mutant and by ~91-fold for MΨ Triple relative to WT Ψ based on the *K*_d(1M)_ (Table 3). Thus, while the RSV MΨ A197G and UGCG to GAGA mutations lead to a loss in NC binding affinity [44], these mutations had no effect on specific recognition of MΨ by RSV Gag. However, the RSV MΨ GG to CC and MΨ Triple mutants, which significantly reduce gRNA packaging in cell culture [30], also led to a loss in specific recognition by RSV Gag in vitro. While we have identified RSV MΨ mutants associated with a loss in Gag specificity, none of these mutations reduced RNA binding specificity to levels associated with the completely non-Ψ RNA 167 (~2000-fold loss in specificity, Figure 4 and Table 3).

To better understand how the mutations to RSV MΨ may affect recognition by Gag, the global conformation of the RNA variants was probed by native polyacrylamide gel electrophoresis (PAGE). Mfold analysis [60] of MΨ A197G and MΨ UGCG to GAGA predicted that these mutations would not significantly alter the secondary structure of these RNAs. In contrast, Mfold predicted that the MΨ GG to CC and the MΨ Triple mutations would alter the RNA secondary structure. The WT RSV MΨ RNA displayed a major, slower-migrating band and two more minor faster migrating bands (Figure S2). A similar band pattern was observed with RSV MΨ A197G and RSV MΨ GG to CC, except that the middle band was not as dominant and the other two bands were more equivalent. RSV MΨ triple and RSV MΨ UGCG to GAGA displayed predominantly the slowest migrating band at lower intensity. Because equal amounts of RNA were loaded, the lower intensities of the bands suggest a more heterogeneous mix of conformations, especially for RSV MΨ Triple. Although not definitive, these results suggest that disruption of the global fold of Ψ may contribute to the reduced specificity of RSV Gag∆PR interaction.

### 3.4. Mutation of HIV-1 Ψ Residues Required for NC Binding Reduce Specific Recognition by HIV-1 Gag∆p6

**Figure 5:**
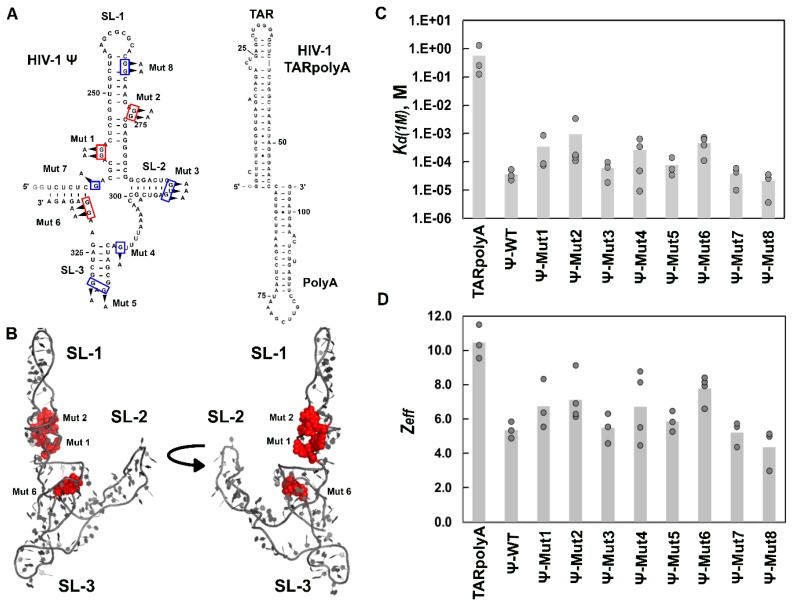
HIV-1 RNA constructs used in this work and salt-titration binding results with HIV-1 Gag∆p6. (**A**) Predicted secondary structures of HIV-1 Ψ (**left**) and HIV-1 TARpolyA (**right**) are shown. Eight mutant Ψ RNAs are indicated by the boxed nt and arrows (Mut1-Mut8). Additional 5’ G nt not present in the HIV-1 genome were added to facilitate in vitro transcription and are shown in gray; (**B**) The location of Mut 1, 2, and 6 mapped onto the all-atom model of HIV-1 Ψ previously determined from small angle x-ray scattering data and computational modeling [61]. Colors in (**A**) and (**B**) indicate mutations that had no effect on specificity (blue), and significant effects (red) based on salt-titration binding assays. Bar graphs of *K*_d(1M)_ values (**C**) and *Z*_eff_ values (**D**) determined from salt-titration assays with HIV-1 Gag∆p6 and HIV-1 TARpolyA, Ψ RNA, and Ψ RNA mutants. Values of three or four trials performed in each case are shown with the height of the bar indicating the mean value.

In agreement with our previous work [27], HIV-1 Gag∆p6 binding to WT Ψ RNA was characterized by an ~15,000-fold greater specificity (*K*_d(1M)_ = 3.6 × 10^−5^ M) and ~six fewer electrostatic interactions (*Z*_eff_ ~ 5) than binding to TARpolyA (*K*_d(1M)_ = 5.6 × 10^−1^ M and *Z*_eff_ ~ 11) (Figure 5C,D, and Table 3). HIV-1 Gag∆p6 exhibited only a minor loss in specificity upon mutation of residues in the loops of SL2 and SL3 of HIV-1 Ψ (Mut 3 and 5; *K*_d(1M)_ ~ 6–8 × 10^−5^ M and relative specificity of 0.47–0.61 of Ψ-WT), and only a modest increase in the number of electrostatic charges mediating the interaction (*Z*_eff_ ~ 5–6) (Figure 5C,D, and Table 3). Based on the partial overlap of the parameter values measured for these mutants with those for Ψ-WT, we concluded that these ~two-fold differences were not significantly different from WT. Similarly, although a point mutation in the single-stranded region between SL2 and SL3 (Mut 4) appeared to lead to a greater loss in specificity (*K*_d(1M)_ ~ 3 × 10^−4^ M; relative specificity of 0.14), based on the variability in the data generated using this mutant, we concluded that it was not significantly different from Ψ-WT. In contrast, a significant loss in specificity was observed upon mutation of residues in the bulge regions of SL1 (Mut 1 and 2), as well as in the region downstream of SL3 (Mut 6) (relative specificity of 0.04–0.11). The final two HIV-1 Ψ mutants tested (Mut 7 and 8) bound HIV-1 Gag∆p6 with a relative specificity of 0.97–1.7 and interacted with a similar number of electrostatic contacts (*Z*_eff_ ~ 4–5) as Ψ-WT (Figure 5C,D, and Table 3). Although we observed different effects of the HIV-1 Ψ mutants on Gag binding specificity, no single Ψ mutant diminished specificity to the level of non-Ψ RNA.

To determine whether the Ψ mutations disrupted the global RNA fold, all HIV-1 Ψ RNA variants were subjected to native-PAGE analysis. The majority of RNAs migrated as a single band corresponding to the RNA dimer, and there were no changes in the global conformation of the mutants relative to Ψ-WT (Figure S2). The only exception was Mut 8, which was a mix of dimer (major band) and monomer (minor band). These results suggest that changes to the global RNA fold and/or dimerization state cannot explain the reduced specificity of HIV-1 Gag∆p6 for the mutant RNAs.

## 4. Discussion

### 4.1. RSV and HIV-1 Gag Share a MA-Dependent Mechanism to Discriminate Ψ from Non-Ψ RNA

In the present work, we have shown that, as for HIV-1 Gag, specific recognition of Ψ RNA by RSV Gag involved a significantly greater nonelectrostatic binding component than interaction with non-Ψ RNA, and binding to Ψ was mediated primarily by the NC domain. In contrast, non-Ψ RNA interactions were characterized by increased electrostatic binding interactions, consistent with binding of both the NC and MA domains. A recent small-angle X-ray scattering (SAXS) and neutron reflectometry study showed that RSV Gag is likely sampling an ensemble of conformations in solution (elongated and bent), but was more compact when interacting with anionic membranes [62]. This suggests that, as previously reported for HIV-1 Gag [10,63,64], RSV Gag may also be extremely flexible, and binding to different RNAs may shift the equilibrium toward different conformational states. We also observed that the MA domain was required for RSV Gag-mediated discrimination of Ψ and non-Ψ RNA, even though MA alone did not display specific binding. We have previously shown that the MA domain plays a similar role in the context of HIV-1 Gag [27].

The lack of direct MA-Ψ RNA interactions in the case of HIV-1 and RSV Gag is consistent with recent CLIP-seq studies showing a lack of HIV-1 MA–gRNA interactions in cells [8,65]. However, MA–tRNA interactions are observed in cells [8] and may serve other functions including specific PM targeting [66,67]. Our results provide further biochemical support for the CLIP-seq data showing a lack of MA–Ψ interactions, which would leave MA free to interact with other RNAs (such as tRNA) and facilitate virion assembly at the PM. Overall, our study showed that both HIV-1 and RSV MA played primarily a regulatory role in specific gRNA recognition by Gag. The major role of NC in specific gRNA packaging in cells is well established in many retroviral systems [13,14,37], whereas the role of MA appears to be more variable. In deltaretroviruses such as bovine leukemia virus and human T-cell leukemia virus type 1 and type 2, MA has been shown to play a more direct role in gRNA binding and/or packaging [38,68,69]. In the case of HIV-1, NC and MA appear to have somewhat redundant roles, as mutations introduced into nucleic acid binding regions do not severely affect RNA incorporation, whereas mutations in both domains simultaneously abolish gRNA packaging [70,71]. In RSV, deleting MA and substituting it with a myristate moiety to ensure Gag PM localization decreased gRNA packaging by ~three fold [37]. Furthermore, adding a myristate moiety to the intact MA domain to enhance Gag PM localization and subvert Gag nuclear trafficking also resulted in diminished gRNA packaging [72,73]. Taken together, these studies suggest a role for MA in gRNA packaging, which may be mediated by its role in subcellular trafficking of the RSV Gag polyprotein.

We observed an RNA concentration-dependence in the capability of HIV-1 Gag to specifically interact with its cognate Ψ RNA element, with optimal discrimination between Ψ and non-Ψ RNA occurring at 20.5 nM RNA, which corresponds to a 1:20 RNA:Gag ratio (Table S2). To determine whether this finding was due to a concentration-dependent change in the oligomeric state of the RNA, native-PAGE analysis was performed. The results showed that at both the lowest (1.5 nM) and highest (20.5 nM) RNA concentrations examined, HIV-1 Ψ-WT RNA was exclusively dimeric (data not shown). In contrast to HIV-1, we observed the greatest MΨ RNA binding specificity at 1.5 nM RNA (1:200 RNA:Gag) in the case of RSV Gag, which is hypothesized to initially interact with gRNA in the nucleus [72], and RSV Gag remains selective at high RNA concentration (Table S2). Although the observed RNA concentration-dependence on specific binding of HIV-1 Gag could be an artifact of the in vitro assay, it may also be caused by changes in the local RNA fold that could not be detected by native-PAGE analysis. Alternatively, the changes in RNA:Gag ratio might impact the protein’s specific recognition capabilities in a biologically relevant manner. Earlier FA salt-titration binding assays with WT and mutant HIV-1 Gag performed at 30–40 nM RNA concentration showed dramatic effects of NC zinc finger mutations on RNA specificity, consistent with expectations based on cell-based assays [74] and supporting the robustness of the in vitro assay [27]. We speculate that the highly specific HIV-1 Gag binding observed at 20–40 nM Ψ RNA (1:20–1:10 RNA:protein ratio) may reflect the possibility that gRNA recognition initially occurs in the cytoplasm at a relatively low Gag concentration and not at the PM where Gag has a higher local concentration [2,8,75,76]. Clearly, more work is needed to test these ideas.

### 4.2. RSV and HIV-1 MA Domains Are Functionally Equivalent in Facilitating Ψ Binding Specificity But Gag Proteins Differ in Cross-Species Ψ RNA Recognition

We also found that both HIV-1 and RSV Gag were able to accommodate a heterologous MA domain while still retaining the same RNA specificity (Table 2). The functional equivalence of these two MA domains in mediating Ψ-binding specificity is surprising in light of the fact that there are significant differences between the two domains with regard to nucleic acid binding affinity [16,47,58] and membrane binding function [58,62,77,78,79]. HIV-1 MA is myristoylated and requires phosphatidyl inositol (4,5) bisphosphate (PI(4,5)P_2_) for correct Gag PM localization [58,77,80]. While RSV MA is not myristoylated, PI(4,5)P_2_ has been shown to play a role in Gag-PM localization in cells [78,79]. Furthermore, PI(4,5)P_2_ mimetic agents stimulate HIV-1 Gag nucleic acid chaperone activity by interacting with MA [16], whereas they bind to MA but do not effect RSV Gag chaperone function in vitro [47]. To our knowledge, cross-packaging between HIV-1 and RSV Gag proteins and gRNAs has not been investigated in cells. Based on our binding assay results (Table 2), we predict that HIV-1 Gag may be capable of packaging RSV gRNA into nascent virions, whereas the converse would not be true.

### 4.3. Ψ Elements Responsible for Specific Recognition by Retroviral Gag Proteins

Previous salt-titration binding assays investigated a HIV-1 Ψ variant (Ψ-12M) wherein 12 single-stranded G bases in loops and bulges (clustered in six regions) were simultaneously mutated. These regions were proposed to be high-affinity NC binding sites based on a SHAPE footprinting study in which the HIV-1 gRNA was probed in vitro in the presence or absence of bound NC [24]. These mutations were performed using a slightly different Ψ RNA construct than the one used in the present work. Nevertheless, we found that HIV-1 Gag binding to the Ψ-12M RNA was characterized by a ~25-fold weaker non-electrostatic binding component relative to WT Ψ RNA [27]. We now report the results of individual mutations at each of these six regions (1–3 mutations per region, see Figure 5A). Mutation of two G-rich bulges in SL1 (Ψ-Mut 1 and 2) and single-stranded G residues downstream of SL3 (Ψ-Mut 6) displayed the largest loss in specificity (Figure 5 and Table 3), with the mutation in the upper single-stranded bulge of SL1 (Mut2) having a similar effect as previously reported for Ψ-12M (~25-fold decrease in specificity). Interestingly, this bulge together with the lower bulge in SL1 (mutation of which reduces specificity ~10-fold in our assay) were found to be the two most statistically significant gRNA NC-interaction sites in the SHAPE study [24]. Our results are also consistent with a recent report identifying the upper bulge in SL1 (i.e., Ψ-Mut 2) as a critical site for full-length HIV-1 Gag interaction in vitro [26]. Mutations at five other locations in Ψ (Mut 3, 4, 5, 7, and 8) resulted in Gag binding specificity that was not distinguishable from Ψ-WT. Consistent with our results for Ψ-Mut 3, previous gRNA packaging studies found SL2 to be less important than the other elements of Ψ in directing gRNA incorporation into virions [8,14,25,26,81,82]. The Ψ-Mut 5 results were more surprising, as most studies agree on the importance of SL3 in packaging [14,82,83,84,85]. Importantly, mutation of a single-stranded G residue within Ψ that had not previously been implicated as a site of NC interaction (Ψ-Mut 7) did not negatively affect Gag binding specificity. Ψ-Mut 8 was the only HIV-1 RNA mutant tested to modestly disrupt dimerization activity (Figure S2B), but consistent with our early report [27], this mutation did not impact Gag binding specificity to this ~100-nt construct. Although a recent mutational interference mapping study examining the RNA residues critical for HIV-1 Gag binding to Ψ did identify the region corresponding to Ψ-Mut 8 as important [86], other studies are more consistent with a lack of direct Gag/NC binding in this region [8,24].

We mapped the three most important sets of residues for promoting specific Gag binding onto a tertiary structural model of HIV-1 Ψ RNA generated by SAXS (Figure 5B) [61]. Interestingly, these mutations cluster together in the central part of the RNA. Because no single-site mutant reduces Gag binding specificity to the level of TARpolyA, we propose that the clustering of several Gag interaction sites in close proximity is an important determinant of specific binding. It is tempting to speculate that in the dimeric form of Ψ, the resulting cluster of six critical interaction sites would provide a unique binding platform for a Gag hexamer, possibly acting as the trigger for virion assembly and thus ensuring packaging of a gRNA dimer [87].

Less is known about the specific RSV Ψ elements that are critical for gRNA packaging. However, two previous mutations (G214C/G215C and MΨ Triple) shown to reduce gRNA packaging efficiency by ~20- and ~50-fold, respectively [30], were tested here and both mutants significantly reduced specific RSV Gag binding in our in vitro assay (~seven- to ~90-fold, respectively). Both of these mutations were predicted to disrupt local secondary structural elements, and in the earlier report, compensatory mutations that restored base pairing rescued packaging to near WT levels [30]. Thus, the authors concluded that secondary structure in this region rather than primary sequence is required for efficient packaging. Native gel electrophoresis carried out here, confirmed a loss of native-like fold for these two mutants (Figure S2A). Mutations at two additional sites previously identified as critical for NC binding (UGCG to GAGA in the SLC tetraloop and A197G) [44,45] did not negatively impact Gag binding selectivity in our in vitro assay. Differences in both the protein and RNA constructs may account for this discrepancy. In addition, although mutations at these sites were shown to compromise infectivity [45], a specific effect on gRNA packaging was not reported. In fact, mutation of the two central residues of the tetraloop (GC to UU) did not significantly affect gRNA packaging [30].

In summary, in this work, we used an FA-based salt-titration assay to investigate binding of RSV Gag to MΨ and non-Ψ RNAs, revealing significant RNA-dependent differences in the binding mode. RSV Gag interacted with MΨ RNA preferentially using only the NC domain, while the increased electrostatic interactions observed with non-Ψ RNA are consistent with simultaneous binding of both NC and MA. Moreover, the presence of MA was required for optimal discrimination of Ψ and non-Ψ RNAs. Similar results were previously obtained in the case of HIV-1 Gag binding to its cognate Ψ RNA versus a non-Ψ RNA [27]. Thus, the overall strategy of Ψ recognition involving direct nonelectrostatic binding the NC domain, and a non-specific but functionally significant contribution from the MA domain, may be more generally conserved across at least a subset of retroviruses. The retroviral MA domains could be swapped without compromising the ability of each Gag protein to recognize its cognate Ψ RNA in a specific manner. Differences were observed in the cross-species recognition capabilities of the retroviral Gag proteins: HIV-1 Gag was able to recognize RSV MΨ specifically, but the converse was not true. Finally, we quantified the contribution of Ψ RNA sequence elements to Gag binding specificity in each system and the results are generally in good agreement with previous in vitro binding and cell-based gRNA packaging assays.

## Figures and Tables

**Table 1:** Binding parameters determined from fluorescence-anisotropy (FA) salt-titration assays with Rous sarcoma virus (RSV) Gag∆PR and human immunodeficiency virus type 1 (HIV-1) Gag∆p6 variants.

**Protein**	**HIV-1 Ψ**		**TARpolyA**	
**HIV-1**	***K*_d(1M)_ (M) ^a^**	***Z*_eff_^b^**	***K*_d(1M)_ (M) ^a^**	***Z*_eff_^b^**
Gag∆p6 ^c^	(5.2 ± 1) × 10^−5^	5.0 ± 0.2	(2.2 ± 1) × 10^−2^	9.1 ± 0.3
CANC ^c^	(2.5 ± 1) × 10^−4^	4.0 ± 0.3	(3.9 ± 2) × 10^−3^	4.7 ± 0.3
	**RSV MΨ**		**RSV 167**	
**RSV**	***K*_d(1M)_ (M) ^a^**	***Z*_eff_^b^**	***K*_d(1M)_ (M) ^a^**	***Z*_eff_^b^**
Gag∆PR	(7.1 ± 5) × 10^−5^	3.7 ± 0.5	(1.3 ± 2) × 10^−1^	7.0 ± 1.2
CANC	(1.3 ± 4) × 10^−5^	2.3 ± 0.2	(4.2 ± 3) × 10^−4^	2.8 ± 0.3
MA	(4.9 ± 7) × 10^+1^	5.8 ± 1.1	(1.9 ± 3) × 10^+2^	5.5 ± 1.4

^a^ *K*_d(1M)_ is the affinity of the specific, nonelectrostatic binding component; ^b^ *Z*_eff_ is the effective charge of the protein-RNA interaction, and reflects the number of Na^+^ ions displaced during binding; ^c^ Data from Webb et al. [27].

**Table 2:** Binding parameters determined from FA salt-titration assays of wild-type (WT) and chimeric Gag constructs.

RNA ^a^		RSV Gag∆PR	H132R	HIV-1 Gag∆p6	R155H
RSV MΨ	*K*_d(1M)_ (M) ^b^	(7.1 ± 5) × 10^−5^	(7.2 ± 3) × 10^−5^	(5.5 ± 2) × 10^−5^	(6.7 ± 3) × 10^−5^
	*Z*_eff_^b^	3.7 ± 0.5	4.7 ± 0.5	4.8 ± 0.2	4.5 ± 0.4
RSV 167	*K*_d(1M)_ (M) ^b^	(1.3 ± 2) × 10^−1^	(1.9 ± 3) × 10^0^	(1.0 ± 1) × 10^−2^	(2.3 ± 2) × 10^−1^
	*Z*_eff_^b^	7.0 ± 1.2	8.9 ± 2.3	8.4 ± 1.0	9.9 ± 1.3
HIV-1 Ψ	*K*_d(1M)_ (M) ^b^	(3.2 ± 5) × 10^−2^	(1.9 ± 3) × 10^−2^	(3.6 ± 2) × 10^−5^	(3.1 ± 1) × 10^−5^
	*Z*_eff_^b^	5.9 ± 1.1	7.1 ± 2.2	5.4 ± 0.5	4.1 ± 0.4
TARpolyA	*K*_d(1M)_ (M) ^b^	(4.0 ± 2.7) × 10^−2^	(2.9 ± 2) × 10^−1^	(5.6 ± 6) × 10 ^−1^	(2.6 ± 3) × 10^0^
	*Z*_eff_^b^	6.2 ± 0.3	7.9 ± 1.2	10.5 ± 1.0	9.2 ± 0.7

^a^ RNA concentrations were 1.5 nM for RSV Gag∆PR and H132R and 20.5 nM for HIV-1 Gag∆p6 and R155H; ^b^ *K*_d(1M)_ and *Z*_eff_ are defined in the legend to Table 1.

**Table 3:** Binding parameters determined from FA salt-titration assays of WT Gag proteins with non-Ψ RNA, WT Ψ, and mutant Ψ RNAs.

	RSV Gag∆PR		
**RNA ^a^**	***K*_d(1M)_ (M) ^b^**	***Z*_eff_^b^**	**Relative Specificity ^c^**
RSV 167	(1.3 ± 2) × 10^−1^	7.0 ± 1.2	5.5 × 10^−4^
RSV MΨ-WT	(7.1 ± 5) × 10^−5^	3.7 ± 0.5	1.0
RSV MΨ UGCG to GAGA	(1.2 ± 0.5) × 10^−5^	2.6 ± 0.2	5.9
RSV MΨ A197G	(4.7 ± 2) × 10^−5^	3.2 ± 0.2	1.5
RSV MΨ GG to CC	(4.9 ± 4) × 10^−4^	4.6 ± 0.5	0.14
RSV MΨ Triple	(6.5 ± 11) × 10^−3^	5.0 ± 1.7	0.011
**HIV-1 Gag∆p6**			
TARpolyA	(5.6 ± 6) × 10^−1^	10.5 ± 1.0	6.4 × 10^−5^
Ψ-WT	(3.6 ± 2) × 10^−5^	5.4 ± 0.5	1.0
Ψ-Mut1	(3.4 ± 5) × 10^−4^	6.7 ± 1.4	0.11
Ψ-Mut2	(9.5 ± 16) × 10^−4^	7.1 ± 1.4	0.038
Ψ-Mut3	(5.9 ± 4) × 10^−5^	5.5 ± 0.9	0.61
Ψ-Mut4	(2.6 ± 3) × 10^−4^	6.7 ± 2.1	0.14
Ψ-Mut5	(7.6 ± 6) × 10^−5^	5.9 ± 0.6	0.47
Ψ-Mut6	(4.6 ± 3) × 10^−4^	7.8 ± 0.8	0.078
Ψ-Mut7	(3.7 ± 2) × 10^−5^	5.2 ± 0.7	0.97
Ψ-Mut8	(2.1 ± 2) × 10^−5^	4.4 ± 1.2	1.7

^a^ RNA concentrations were 1.5 nM for RSV Gag∆PR and 20.5 nM for HIV-1 Gag∆p6; ^b^ *K*_d(1M)_ and *Z*_eff_ are defined in the legend to Table 1; ^c^ Specificity of the WT Ψ RNAs was set to 1.0 and the relative specificity of the non-Ψ or mutant Ψ RNAs was calculated as *K*_d(1M)_ (Ψ-WT)/*K*_d(1M)_ (Ψ-variant).

## Supplementary Materials

The following supplementary figures are available online at www.mdpi.com/1999-4915/8/9/256/s1, Figure S1: Bar graphs showing *K*_d(1M)_ values and *Z*_eff_ values determined from salt-titration assays using HIV-1 Gag∆p6 with HIV-1 Ψ and HIV-1 TARpolyA, and RSV Gag∆PR with RSV MΨ and RSV 167, Figure S2: Native-PAGE of fluorescently-labeled WT and mutant RSV MΨ (A) and HIV-1 Ψ (B) RNAs, Table S1: Apparent binding dissociation constants determined from FA assays for WT RSV GagΔPR, RSV Gag protein variants, WT HIV-1 GagΔp6, and the H132R chimera binding to RSV MΨ and RSV 167 RNAs, Table S2: Binding parameters determined from FA salt-titration assays with WT RSV Gag∆PR and HIV-1 Gag∆p6 at varying RNA concentrations, Table S3: Apparent dissociation constants determined from FA assays for WT RSV GagΔPR binding to WT and mutant RSV MΨ, RSV 167, HIV-1 Ψ and HIV-1 TARpolyA RNAs.
