# Supplementary material for: Functional Equivalence of Retroviral MA Domains in Facilitating Psi RNA Binding Specificity by Gag
Source: Viruses. 2016 Sep 19;8(9):256. doi: 10.3390/v8090256 (PMC5035970; doi:10.3390/v8090256)
Supplement: Supplementary file 1 [file viruses-08-00256-s001.pdf]

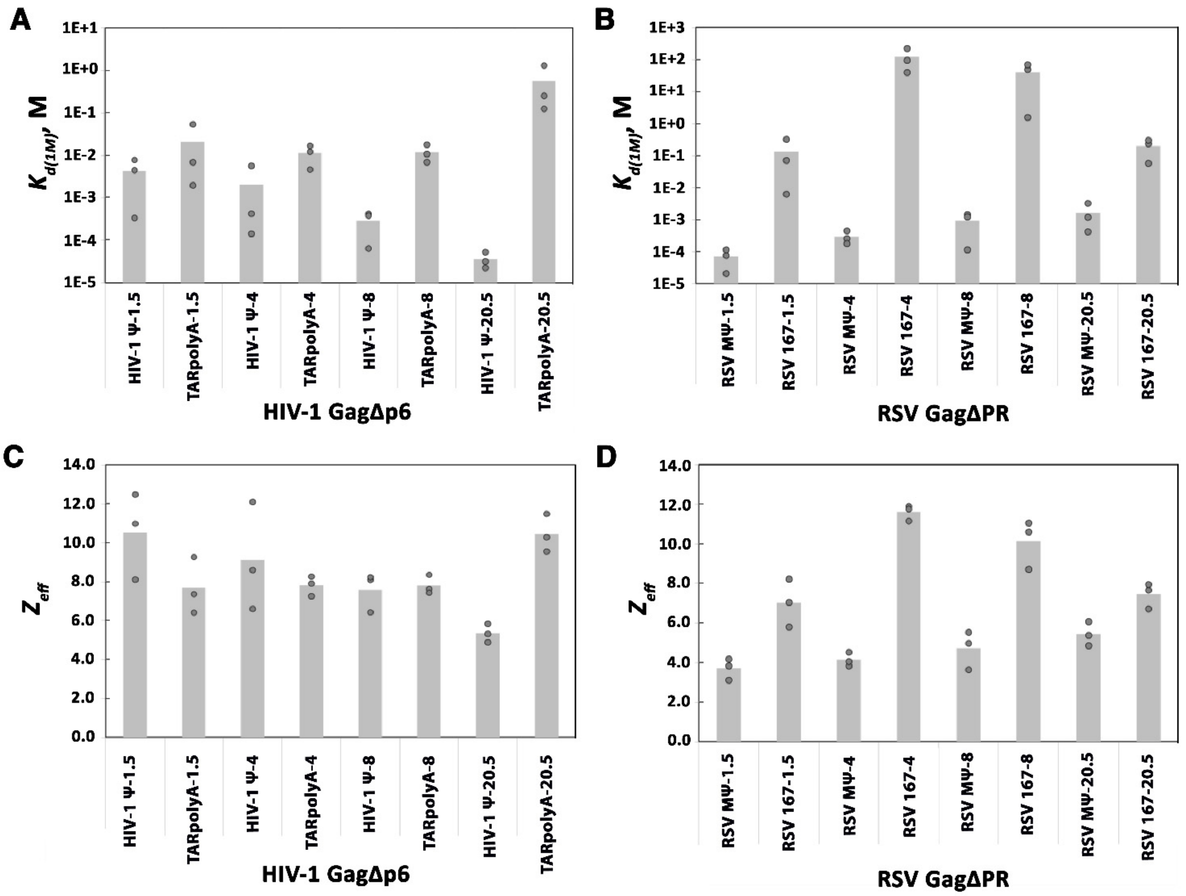

Figure S1. Bar graphs showing $K_{d(1M)}$ values and Z_{eff} values determined from salt-titration assays using HIV-1 GagΔp6 with HIV-1 Ψ and HIV-1 TARpolyA (A, C), and RSV GagΔPR with RSV MΨ and RSV 167 (B, D). RNA concentrations range from 1.5 nM to 20.5 nM, as indicated by RNA-concentration in nM. Values of three trials performed in each case are shown with the height of the bar indicating the mean value.

Table S1. Apparent binding dissociation constants determined from FA assays for WT RSV GagΔPR, RSV Gag protein variants, WT HIV-1 GagΔp6, and the H132R chimera binding to RSV MΨ and RSV 167 RNAs.

	**Dissociation Constant, K_d (nM)[a]**	
**Protein Variant**	**RSV MΨ**	**RSV 167**
RSV GagΔPR	15 ± 4	18 ± 0.6
RSV CANC	11 ± 2	21 ± 6
RSV GagΔNC[b]	1,421 ± 393	5,837 ± 1,269
RSV MA	1,744 ± 614	5,945 ± 598
HIV-1 GagΔp6	14 ± 6	13 ± 3
H132R	13 ± 2	20 ± 4

[a] Binding was conducted in 20 mM HEPES pH 7.5, 1 mM $MgCl_2$, and 50 mM monovalent ions and RNA was at 1.5 nM. Protein concentrations ranged from 0 to 1.84 μM for RSV GagΔPR and H132R, from 0 to 1.85 μM for RSV CANC and HIV-1 GagΔp6, from 0 to 6.80 μM for RSV GagΔNC, and from 0 to 24.9 μM for RSV MA. Reported values are an average of at least three measurements with the standard deviation indicated.

[b] To generate the plasmid encoding RSV GagΔNC, primers were designed to amplify DNA encoding leucine 385 (in CA) through the last residue of SP (methionine) with two added stop codons from template pET28.(-His).GagΔPR. The product was then subcloned into the SbfI and HindIII sites of pET28.(-His).GagΔPR, replacing the original C terminus of CA and NC with the C terminus of CA followed by stop codons, thus generating pET28. (-His).GagΔNC. RSV GagΔNC was expressed in BL21(DE3)-pRIL cells in autoinduction media. Cells were sonicated in lysis buffer (25 mM HEPES, pH 7.5, 100 mM NaCl, 0.1 mM EDTA, 0.1 mM TCEP, 0.1% NP-40, rLysozme, benzonase and Roche Complete protease inhibitors). The cell debris was removed by centrifugation at 21,000 x g for 30 min. The supernatant was loaded onto a cation exchange column (Poros 50 HS) and the protein eluted in a 15 column volume linear gradient of NaCl from 0.1-1 M in 25 mM HEPES, pH 7.5, 0.1 mM EDTA, and 0.1 mM TCEP. Fractions containing protein were pooled, concentrated, and purified on a Superdex 200 column (GE Healthcare) in 10 mM HEPES, pH 7.5, 200 mM NaCl, 0.1 mM EDTA, and 0.1 mM TCEP. Concentrated protein was snap frozen in small aliquots in liquid nitrogen and stored at -80 °C. RSV GagΔNC concentration was determined from the absorbance at 280 nm using the molar extinction coefficient: 54,680 $M^{-1} cm^{-1}$.

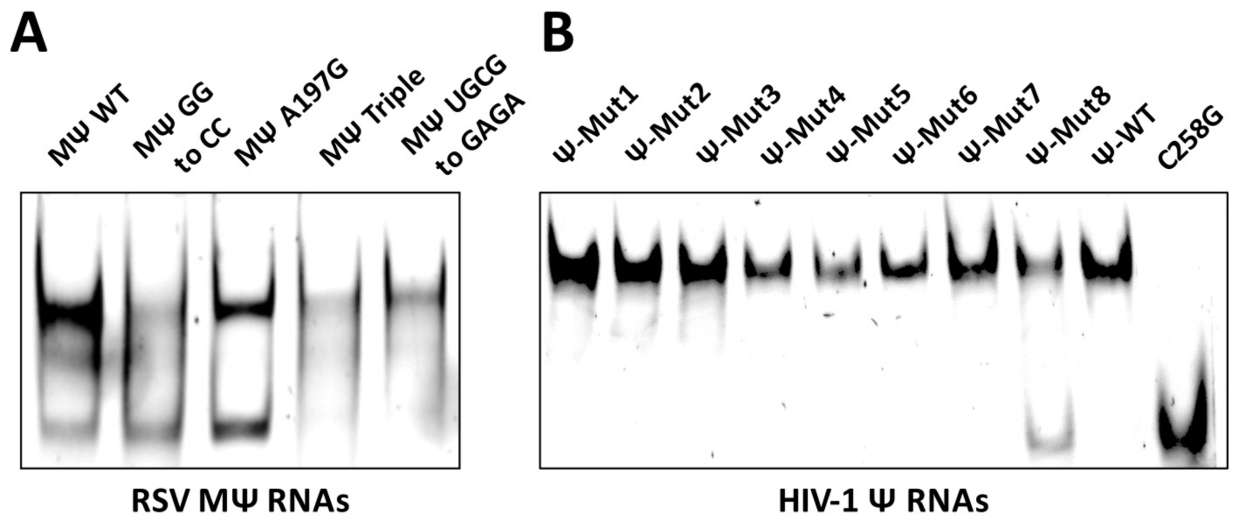

Figure S2. Native-PAGE of fluorescently-labeled WT and mutant RSV MΨ (A) and HIV-1 Ψ (B) RNAs. C258G is a point mutant in the dimerization initiation site of HIV-1 Ψ known to disrupt dimer formation [27]. RNAs were labeled as described in the main text and folded as follows: 1-5 μM RNA in 50 mM HEPES, pH 7.5, was heated at 80°C for 2 min, then 60°C for 2 min, and then $MgCl_2$ was added to a final concentration of 10 mM, followed by incubation on ice for a minimum of 30 min. Folded RNAs were run on 8% native-polyacrylamide gels (19:1 acrylamide: bis-acrylamide) also containing 1 mM $MgCl_2$ and TB (18 mM Tris, 9 mM borate, pH 8) at 4°C in TB running buffer. The final concentration of RSV and HIV-1 RNA run on the gels shown was 50 nM and 20.5 nM, respectively. In the case of RSV, this concentration is much higher than the 1.5 nM concentration used in the salt-titration binding assays; however, a similar band pattern is observed at 1.5 nM [RNA] and the higher concentration is presented here for clarity. Gels were visualized using the fluorescence mode on an AlphaImager EP (AlphaInnotech).

Table S2. Binding parameters determined from FA salt-titration assays with WT RSV GagΔPR and HIV-1 GagΔp6 at varying RNA concentrations.

RNA-Concentration (nM)		RSV GagΔPR	HIV-1 GagΔp6
Cognate Ψ RNA-1.5[b]	$K_{d(1M)}$ (M)[a]	$(7.1 \pm 5) \times 10^{-5}$	$(5.4 \pm 6) \times 10^{-3}$
	Z_{eff} [a]	3.7 ± 0.5	10 ± 2.2
Cognate Non-Ψ RNA-1.5[c]	$K_{d(1M)}$ (M)[a]	$(1.3 \pm 2) \times 10^{-1}$	$(2.1 \pm 3) \times 10^{-2}$
	Z_{eff} [a]	7.0 ± 1.2	7.7 ± 1.5
Cognate Ψ RNA-4[b]	$K_{d(1M)}$ (M)[a]	$(3.0 \pm 1) \times 10^{-4}$	$(2.1 \pm 3) \times 10^{-3}$
	Z_{eff} [a]	4.2 ± 0.4	9.1 ± 2.8
Cognate Non-Ψ RNA-4[c]	$K_{d(1M)}$ (M)[a]	$(1.2 \pm 0.9) \times 10^{+2}$	$(1.1 \pm 0.6) \times 10^{-2}$
	Z_{eff} [a]	11.6 ± 0.4	7.8 ± 0.5
Cognate Ψ RNA-8[b]	$K_{d(1M)}$ (M)[a]	$(9.3 \pm 7) \times 10^{-4}$	$(2.9 \pm 2) \times 10^{-4}$
	Z_{eff} [a]	4.7 ± 1.0	7.6 ± 1.0
Cognate Non-Ψ RNA-8[c]	$K_{d(1M)}$ (M)[a]	$(4.0 \pm 4) \times 10^{+1}$	$(1.2 \pm 0.6) \times 10^{-2}$
	Z_{eff} [a]	10.1 ± 1.2	7.8 ± 0.5
Cognate Ψ RNA-20.5[b]	$K_{d(1M)}$ (M)[a]	$(1.6 \pm 1) \times 10^{-3}$	$(3.6 \pm 2) \times 10^{-5}$
	Z_{eff} [a]	5.4 ± 0.6	5.4 ± 0.5
Cognate Non-Ψ RNA-20.5[c]	$K_{d(1M)}$ (M)[a]	$(2.0 \pm 1) \times 10^{-1}$	$(5.6 \pm 6) \times 10^{-1}$
	Z_{eff} [a]	7.5 ± 0.6	10.5 ± 1.0

[a] $K_{d(1M)}$ and Z_{eff} are defined in the legend to Table 1.

[b] Cognate Ψ RNA is HIV-1 Ψ for HIV-1 GagΔp6 and RSV MΨ for RSV GagΔPR.

[c] Cognate non-Ψ RNA is TARpolyA for HIV-1 GagΔp6 and RSV 167 for RSV GagΔPR.

Table S3. Apparent dissociation constants determined from FA assays for WT RSV GagΔPR binding to WT and mutant RSV MΨ, RSV 167, HIV-1 Ψ and HIV-1 TARpolyA RNAs.

	**Dissociation Constant, K_d (nM)[a]**
**RNA Variant**	**RSV GagΔPR**
RSV MΨ WT	15 ± 4
RSV 167	18.0 ± 0.6
RSV MΨ UGCG to GAGA	13 ± 2
RSV MΨ A197G	13 ± 4
RSV MΨ GG to CC	15 ± 6
RSV MΨ Triple	18 ± 10
HIV-1 Ψ	14 ± 2
HIV-1 TARpolyA	18 ± 6

[a] Binding was conducted in 20 mM HEPES, pH 7.5, 1 mM $MgCl_2$, and 50 mM monovalent ions, and RNAs were at 1.5 nM. Protein concentration ranged from 0 to 1.84 μM. Reported values are an average of at least three measurements with the standard deviation indicated.
